# The Study of Overexpression of Peroxiredoxin-2 Reduces MPP^+^-Induced Toxicity in the Cell Model of Parkinson’s Disease

**DOI:** 10.1007/s11064-023-03880-5

**Published:** 2023-02-18

**Authors:** Menghao Liu, Shuqian Zuo, Xing Guo, Junyu Peng, Yaoping Xing, Yanjie Guo, Chaokun Li, Hongxia Xing

**Affiliations:** 1 Department of Neurology, The First Affiliated Hospital of Xinxiang Medical University, Xinxiang, China; 2 Department of Neurology, The Third Affiliated Hospital of Xinxiang Medical University, Xinxiang, China; 3 School of Basic Medical Sciences, Xinxiang Medical University, Xinxiang, China; 4 Key Laboratory of Movement Disorders, Xinxiang, China

**Keywords:** Peroxiredoxin-2, ROS, MPP^+^, Transfection, SIRT1

## Abstract

Parkinson’s disease (PD) is a chronic neurodegenerative disorder characterized by dopaminergic neuron loss, which is related to excessive reactive oxygen species (ROS) accumulation. Endogenous peroxiredoxin-2 (Prdx-2) has potent anti-oxidative and anti-apoptotic effects. Proteomics studies revealed plasma levels of Prdx-2 were significantly lower in PD patients than in healthy individuals. For further study of the activation of Prdx-2 and its role in vitro, SH-SY5Y cells and the neurotoxin 1-methyl-4-phenylpyridinium (MPP^+^) were used to model PD. ROS content, mitochondrial membrane potential, and cell viability were used to assess the effect of MPP^+^ in SH-SY5Y cells. JC-1 staining was used to determine mitochondrial membrane potential. ROS content was detected using a DCFH-DA kit. Cell viability was measured using the Cell Counting Kit-8 assay. Western blot detected the protein levels of tyrosine hydroxylase (TH), Prdx-2, silent information regulator of transcription 1 (SIRT1), Bax, and Bcl-2. The results showed that MPP^+^-induced accumulation of ROS, depolarization of mitochondrial membrane potential, and reduction of cell viability occurred in SH-SY5Y cells. In addition, the levels of TH, Prdx-2, and SIRT1 decreased, while the ratios of Bax and Bcl-2 increased. Then, Prdx-2 overexpression in SH-SY5Y cells showed significant protection against MPP^+^ -induced neuronal toxicity, as evidenced by the decrease in ROS content, increase in cell viability, increase in the level of TH, and decrease in the ratios of Bax and Bcl-2. Meanwhile, SIRT1 levels increase with the level of Prdx-2. This suggests that the protection of Prdx-2 may be related to SIRT1. In conclusion, this study indicated that overexpression of Prdx-2 reduces MPP^+^-induced toxicity in SH-SY5Y cells and may be mediated by SIRT1.

## Introduction

Parkinson’s disease (PD) is the second most common neurodegenerative disease in the world. PD prevalence increases with age [1]. Morbidity increases by a factor of 5–10 from the sixth to ninth decades of life [2]. There are several different pathogenic mechanisms that contribute to dopaminergic neuronal damage, including oxidative stress, inflammation, mitochondrial dysfunction [3]. In recent years, oxidative stress has been widely implicated in the pathogenesis of a variety of diseases, including stroke, cancer, and neurodegenerative disorders [4–6]. Oxidative stress arises from dysregulation of cellular redox activity where production of reactive oxygen species (ROS) outweighs clearance by endogenous antioxidant enzymes and molecular chaperones [7]. ROS are a byproduct of normal metabolism and are formed in the mitochondria during oxidative phosphorylation. When ROS are produced in excess, they cause oxidative stress, which kills cells by damaging intracellular proteins, DNA, and lipids, leading to the degenerative death of dopaminergic neurons in PD patients [8].

Peroxiredoxins are common family proteins that act as antioxidant enzymes by converting several peroxides, such as hydroperoxides, to water and alcohol, respectively [9]. Peroxiredoxin-2 (Prdx-2) is one of six isoenzymes with redox-active cysteines capable of reducing peroxides. It is abundantly expressed in the brain tissue and is considered to be neuron-specific, where it is expressed in neurons of the cerebral cortex, hippocampus, cerebellum, basal ganglia, substantia nigra, and spinal cord, mainly in the cytoplasm and nucleus but not in glial cells [10]. Prdx-2 is involved in a variety of cellular processes, including cell division, gene expression, differentiation, and apoptosis [11]. As a rich antioxidant enzyme in neurons, Prdx-2 plays a significant role in PD. The previous study discovered that Prdx-2 expression in the plasma is significantly lower in Parkinson’s disease patients [12]. A study has pointed out that decreasing Prdx-2 enzyme activity leads to acceleration of neuronal death caused by increased intracellular ROS generation in PD mouse models and MPP^+^-treated cells [13]. However, the molecular mechanism of Prdx-2 action is poorly defined. An in-depth study of the pathogenesis of Prdx-2 is helpful to discover new potential therapeutic targets.

Silent information regulator of transcription 1 (SIRT1) is a nicotinamide adenine dinucleotide (NAD^+^)-dependent deacetylase. SIRT1 interacts with a variety of transcription factors, including NF-κB, p53, and forehead box subgroup O (FOXOs), as well as proteins involved in DNA repair such as DNA-dependent protein kinase (DNA-PK) [14, 15]. It plays an important role in many biological processes, including oxidative stress, apoptosis and senescence, gene transcription, and metabolism [16].

Zhang et al. [17] found that the functional change of Prdx-2 led to the accumulation of endogenous ROS. ROS activated AMPK, which then phosphorylated SIRT1 and inhibited its deacetylation activity. In an effort to identify whether overexpressed Prdx-2 has a protective effect and affects SIRT1 in PD, the human neuroblastoma cell line SH-SY5Y and transfection with Prdx-2 were used to investigate.

In this study, SH-SY5Y cells were treated with 1-methyl-4-phenylpyridinium (MPP^+^) to create a cellular model approximating PD conditions. Detecting the level of tyrosine hydroxylase (TH) is necessary for determining whether the model is successful. TH is the rate-limiting component in the catecholamine production pathway and transforms tyrosine into L-dopamine, the precursor of dopamine (DA) [18]. Apoptosis [19] and oxidative stress [20] both serve as triggers for MPP^+^-mediated neurotoxicity. So the expression of the apoptosis-related protein markers Bax and Bcl-2 was investigated.

## Methods

### Cell Cultures and Treatment

Human neuroblastoma cell line SH-SY5Y was obtained from ATCC (Shanghai, China). The cells were cultured at 37 °C with 5% CO_2_ by using DMEM high glucose (HyClone, USA) supplemented with 10% FBS (Biological Industries, Israel) and 100 U/ml penicillin as well as 100 ug/ml streptomycin. MPP^+^ (MCE, USA) was then added to SH-SY5Y cells and cultured for 48 h. The MPP^+^ powder was prepared into a 10 mM storage solution following the manufacturer’s instructions. In subsequent experiments, dilute the storage solution with complete cell culture media to achieve the desired concentration.

### Cell Viability

The viability of SH-SY5Y cells was determined using the Cell Counting Kit-8 (CCK8, Dojindo, Japan) assay. Cells were plated at a density of 8000 cells/cm2 in 96-well culture dishes (Corning) and incubated with different concentrations of MPP^+^ (0, 0.2, 0.4, 0.6, 0.8, 1, 2 mM) for 48 h. Subsequently, 10 ml of a solution of CCK8 in culture medium was added to each well, and the incubation continued for an additional 1 h. Finally, cell viability was quantified by measuring the absorbance value at 450 nm, and the results were expressed as percentages of the control.

### Total ROS Measurement

To assess the oxidative stress response of MPP^+^ in SH-SY5Y cells, the reactive oxygen species (ROS) test kit (Beyotime Biotechnology, China) was used. SH-SY5Y cells were cultured with MPP^+^ in 6-well plates for 48 h. After incubation, the medium was removed, and the cells were washed with phosphate-buffered saline (PBS). The plate was then incubated for 45 min at 37 °C in the dark with 2,7-Dichlorodihydrofuoresce in diacetate (DCFH-DA) solution. Flow cytometry was used to evaluate the ROS content.

### Mitochondrial Membrane Potential Measurement

JC-1 (5’, 6, 6’-tetrachloro-1, 1’, 3, 3’-tetraethylbenzimidazolylcarbocyanine iodide) is a fluorescent probe that is frequently used to measure fluorescence shifts in order to assess mitochondrial membrane potential (ΔΨm). When the potential of the mitochondrial membrane is high, JC-1 aggregates in the mitochondrial matrix and forms a polymer that fluoresces red (Ex = 585 nm and Em = 590 nm). When the potential of the mitochondrial membrane is low, JC-1 remains in the monomer state in the matrix that fluoresces green (Ex = 515 nm and Em = 529 nm). The level of mitochondrial depolarization is shown by the red/green fluorescence intensity ratio.

Here, SH-SY5Y cells were treated for 48 h with different concentrations of MPP^+^. Then the cells were washed with PBS and incubated with JC-1 working solution (Solarbio, China) for 20 min at 37 °C in the dark. After that, the JC-1 dye was taken out and the JC-1 buffer was rinsed three times. A confocal microscope was used to capture images of the medium-dwelling cells.

### Western blot

Cells were collected, washed twice with PBS, and lysed in lysis buffer. Lysates were centrifuged at 4 °C, and the supernatant was quantified by the BCA Protein Assay Kit (Beyotime Biotechnology, China). 12.5% SDS-PAGE (sodium dodecyl sulfate–polyacrylamide gels) was utilized to separate the proteins and then transferred onto the polyvinylidene difluoride (PVDF) membrane. The membranes were sealed in 5% defatted milk at room temperature for 1.5 h. Then, the membranes were incubated with antibodies against Prdx-2 (Abcam, Britain, 1:3000), tyrosine hydroxylase (TH, CST, USA, 1:3000), SIRT1 (CST, USA, 1:3000), Bax (CST, USA, 1:5000), and Bcl-2 (CST, USA, 1:5000), followed by incubation with secondary antibodies for one hour at room temperature. The enhanced chemiluminescence (ECL, Millipore, USA) Western blotting detection system was used to visualize the bands. The ImageJ software was used for quantifying the protein with actin (CST, USA, 1:5000) as the internal control.

### Cell Transfection

Plasmid vectors for overexpressing Prdx-2 were constructed by gene pharma (JiKai Gene, Shanghai, China). Cells were plated the day before transfection using DMEM with 10% FBS. Lipofectamine 2000 (Invitrogen, CA, USA) was used for transfection in accordance with the manufacturer’s instructions. The cells were then incubated for 5 h at 37 ℃. Then, the FBS was given until the final concentration in the DMEM was 10%. After transfection, the medium was replaced with a complete medium containing MPP^+^ (1.2 mM). Transfected cells were utilized for subsequent experiments after 48 h.

### RT-qPCR

After collecting the transfected cells, RT-qPCR was utilized to assess the transfection effectiveness of Prdx-2. Using the Trizol reagent (Ambion, USA) to extract the total RNA, the cDNA was synthesized with the RNA Transcription Kit (Novoprotein, China). The SYBR Green RT-qPCR apparatus was then applied to perform RT-qPCR. U6 was considered as internal control. Primer sequences were designed using Primer Express software (Applied Biosystems), and the sequences were listed as follows: Prdx-2 (forward: 5’-CCCACCTGGCTTGGATCAAC-3’, reverse: 5’-CAGTGATCTGGCGAAGGACA-3’). U6 (forward: 5’-GGAACGATACAGAGAAGATTAGC-3’, reverse: 5’-TGGAACGCTTCACGAATTTGCG-3’). The relative gene expression was calculated using the 2^−ΔΔCt^ technique.

### Data Analysis

All data were analyzed using GraphPad 8.0 software. The data were presented as means ± SD. Students’ t-test was used to calculate the differences between two groups. The one-way ANOVA was used to analyze comparisons between multiple groups. *P* < 0.05 was regarded as statistically significant.

## Results

### MPP^+^-induced Accumulation of ROS in SH-SY5Y Cells and Decreased cell Viability

**Fig. 1:**
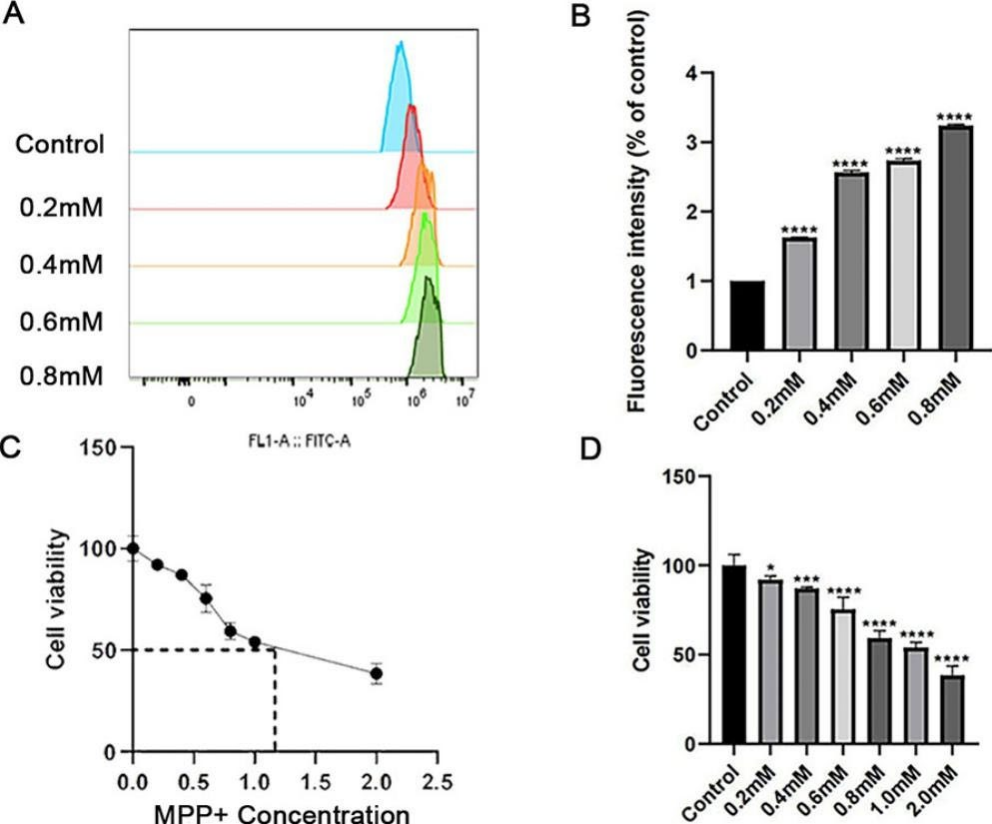
The relative ROS level and the cell viability in each group were compared to the control group. (**A)** The images of ROS content were obtained by flow cytometry using DCFH-DA as a probe. The x-axis represents the FITC-A channel, and the y-axis represents the cell counts. (**B**) The related quantitative analysis of the total ROS measurement result showed intracellular ROS levels in the SH-SY5Y cells at different MPP^+^ concentrations. (**C**) CCK8 documented that cell viability was decreased by MPP^+^ and that the IC50 of MPP^+^ was 1.233 mM. (**D**) The statistical results of cell viability at different MPP^+^ concentrations compared to the control group were shown. *P < 0.05; ***P < 0.001; ****P < 0.0001

MPP^+^ incubation times and concentrations were set up based on the previous experiments. SH-SY5Y cells were treated with 0, 0.2, 0.4, 0.6, 0.8, 1, and 2 mM for 48 h to increase ROS levels in cells. The results showed that ROS production increased with exposure to high concentrations of MPP^+^. ROS are byproducts of normal metabolism in most cell types in the body. From a concentration of 0.2 mM, ROS accumulation increased statistically significantly compared with the normal cell group (*P* < 0.0001) (Fig. 1A and B, n = 3). It is well known that high ROS levels may affect neurons and result in neuronal death. CCK8 was used to assess cell viability, and the results revealed that MPP^+^ caused a dose-dependent decrease. At a concentration of 0.6 mM, MPP^+^ decreased the cell viability by 25% (*P* < 0.0001). Moreover, the IC50 value of MPP^+^ on SH-SY5Y cells was 1.233 mM (Fig. 1C and D, n = 5).

### MPP^+^-induced Mitochondria Membrane Depolarization

**Fig. 2:**
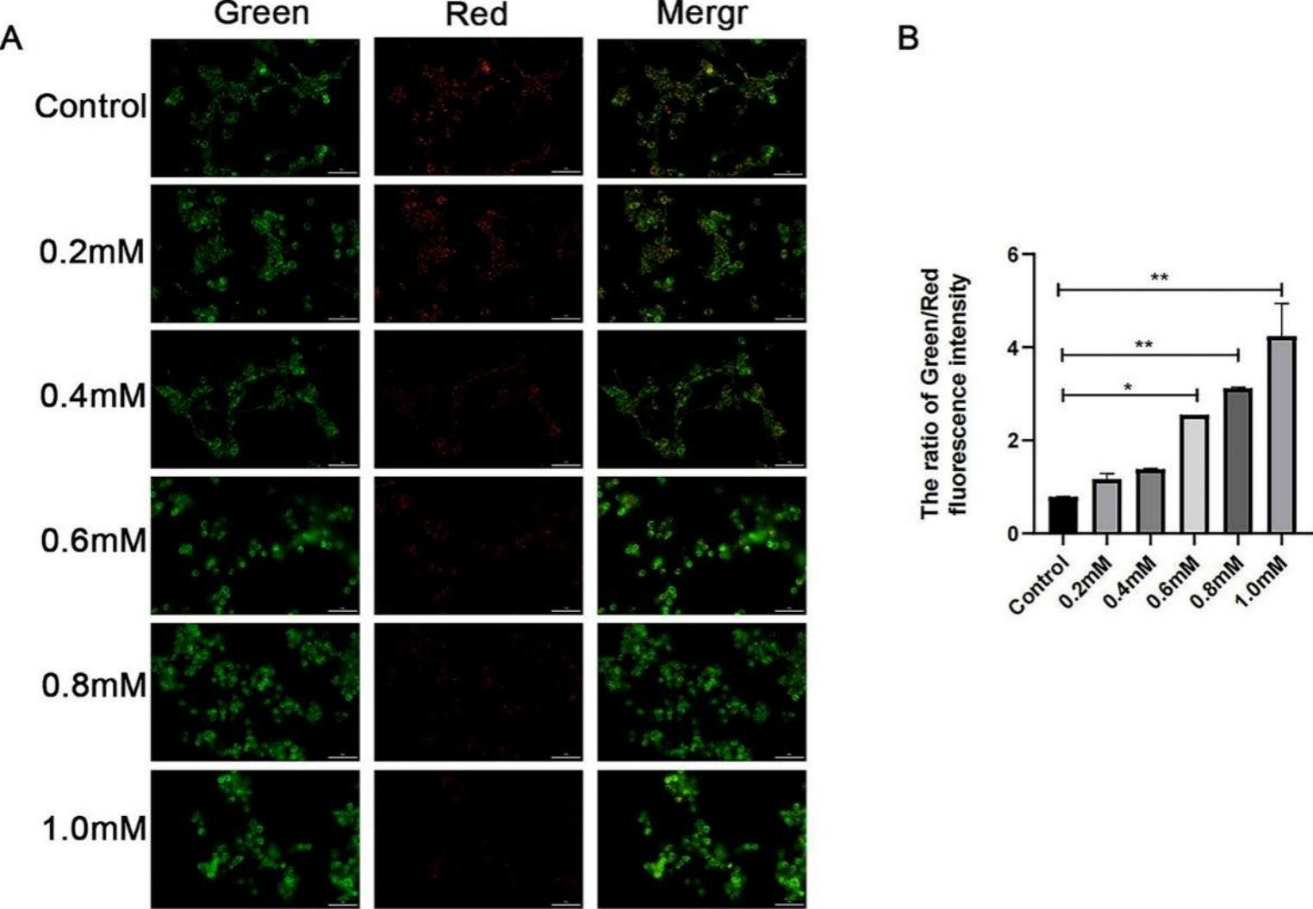
MPP^+^-induced mitochondrial membrane depolarization. (**A**) The red fluorescence intensity decreased with concentrations, and the green fluorescence intensity increased correspondingly. Scale bars: 50 μm. (**B**) The quantity of greenness and red staining in each individual picture was measured. When treated with 0.6 mM, the green/red ratio was statistically significant. *P < 0.05; **P < 0.01

It is known that mitochondria are both generators and targets of ROS. The mitochondrial respiration chain generates ROS, and ROS (either endogenous or exogenous) can impair mitochondrial function. An indication of the mitochondrial state is the mitochondrial membrane potential. The mitochondrial membrane potential (MMP) after MPP^+^ application was detected by the JC-1 assay. As shown in Fig. 2A, compared with the 0 mM group, the red fluorescence intensity decreased with concentrations and the green fluorescence intensity increased correspondingly. The quantity of greenness and red staining in each individual picture was measured using ImageJ software. When treated with 0.6 mM, compared to the control, the green/red ratio was statistically significant. (*P* < 0.05, Fig. 2B).

### MPP^+^ Decreased TH, Prdx-2, SIRT1 Protein Levels and Increased the Ratio of Bax / Bcl-2

**Fig. 3:**
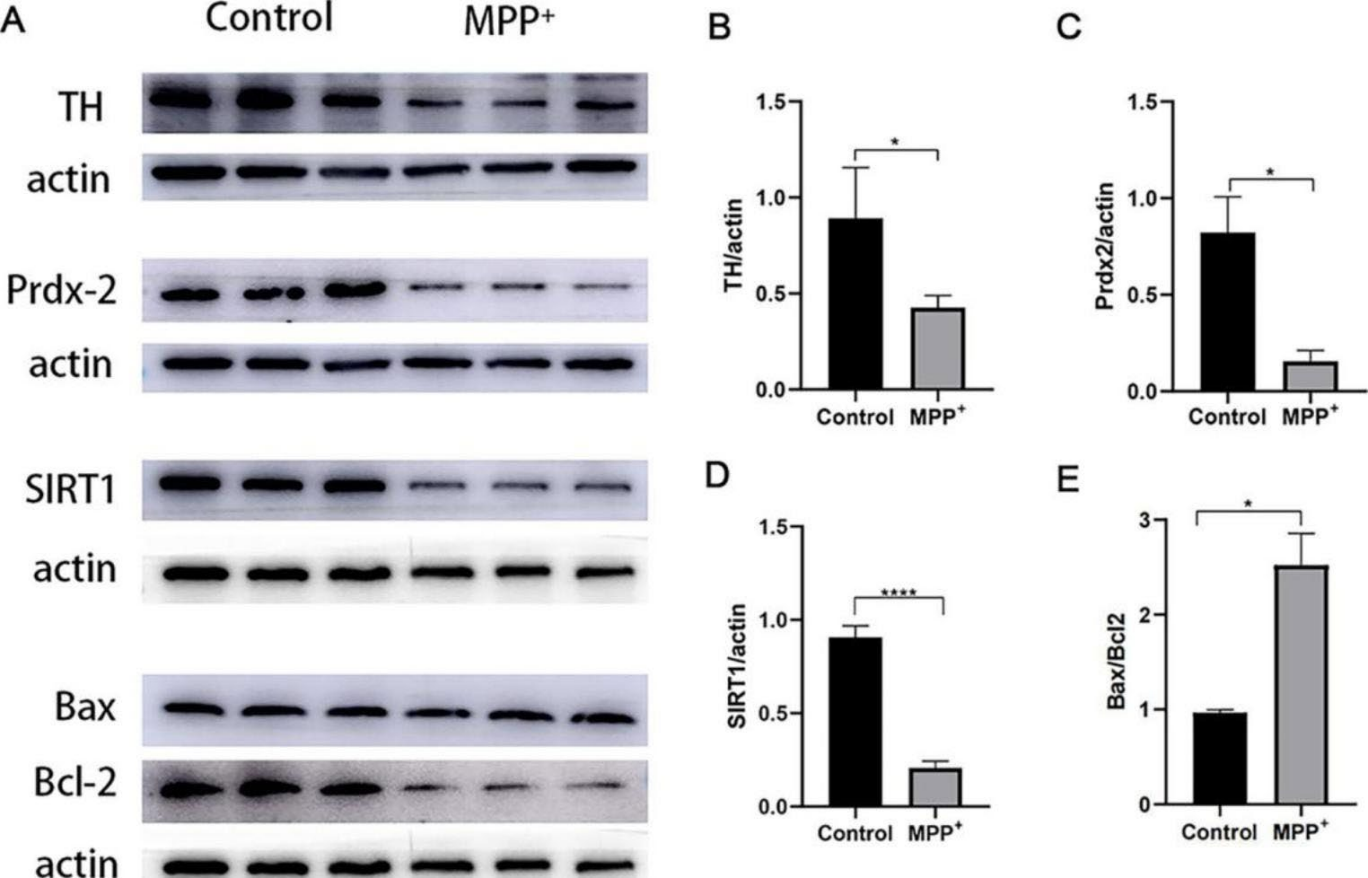
(**A**) The representative band and the density of proteins from SH-SY5Y cells treated with MPP^+^. (**B**) Densitometric analysis of protein expression of TH. (**C**) Densitometric analysis of protein expression of Prdx-2. (**D**) Densitometric analysis of protein expression of SIRT1. (**E**) Densitometric analysis of protein expression of Bax/Bcl-2.

The effects of MPP^+^ were investigated on SH-SY5Y cells after 48 h of treatment with 1.2 mM of MPP^+^. TH, the rate-limiting enzyme in the synthesis of dopamine, was used as a marker for cell function. Western blot results showed that there was a lower expression of TH in the MPP^+^ group than in the control group (*P* = 0.0416, n = 3) (Fig. 3A and B). This finding implies that the ability of SH-SY5Y cells to synthesize dopamine was reduced. This study was designed to examine the role of Prdx-2, so the expression of Prdx-2 in SH-SY5Y cells was analyzed. The outcome showed that MPP^+^ decreased Prdx-2 levels significantly (*P* = 0.0264, n = 3) (Fig. 3A and C). At the same time, the MPP^+^ group has a lower expression of SIRT1 (*P* < 0.0001) (Fig. 3A and D). Furthermore, MPP^+^ increased the ratio of Bax to Bcl-2 (*P* = 0.0097, n = 3) (Fig. 3A and E).

### Overexpression of Prdx-2 Reduced the Production of ROS Induced by MPP^+^

**Fig. 4:**
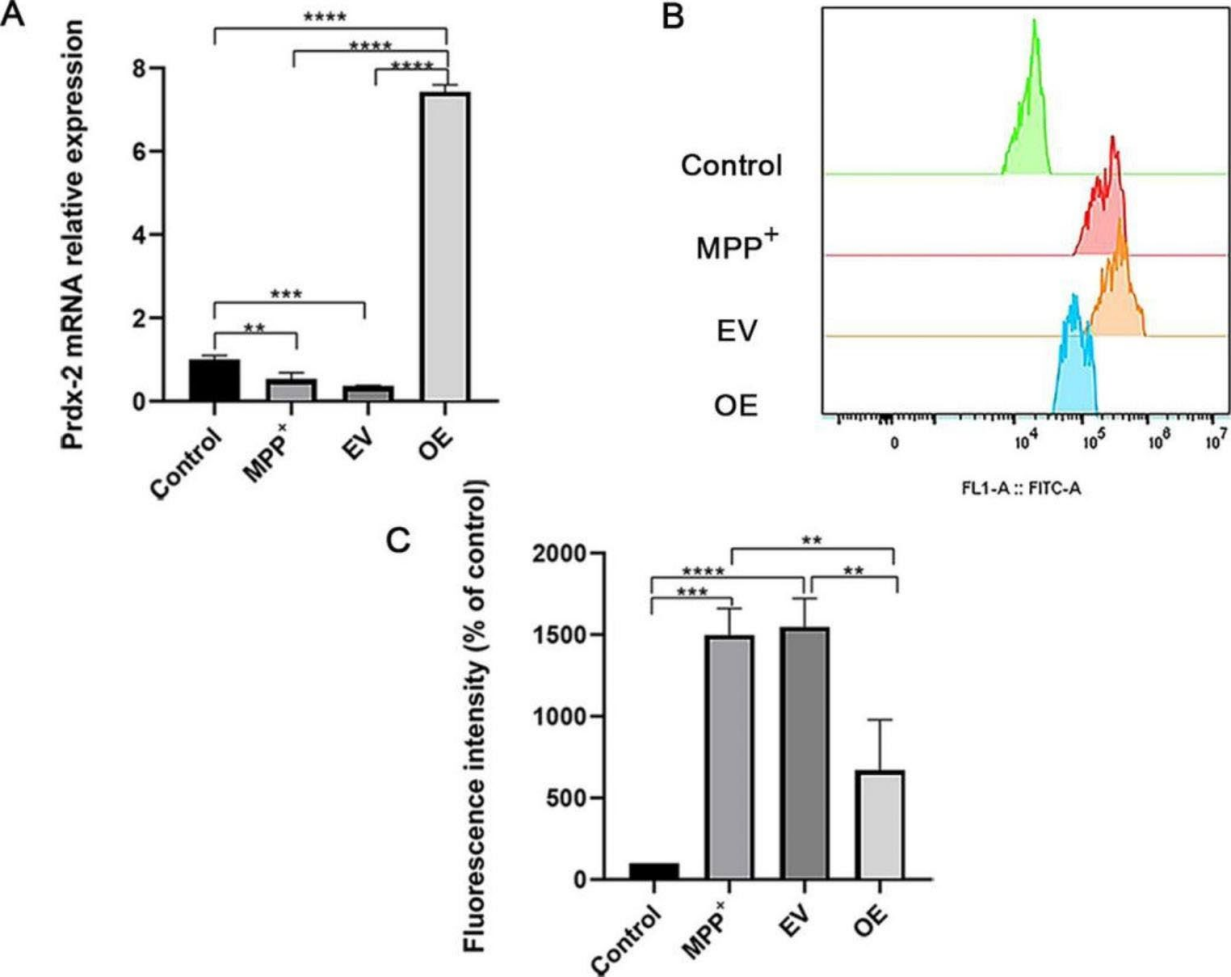
Overexpression of Prdx-2 reduced the production of ROS induced by MPP^+^. (**A**) Quantitative RT-qPCR validates the mRNA expression of Prdx-2. (**B**) The images of ROS levels were determined by flow cytometry using DCFH-DA as a probe after cells were transfected with the Prdx-2 plasmid, and the x-axis represents the FITC-A channel and the y-axis represents the cell counts. (**C**) The quantitative analysis of total ROS measurement in four groups showed a lower ROS level in the OE group than in the MPP^+^ group and the EV group. **P < 0.01; ***P < 0.001; ****P < 0.0001

SH-SY5Y cells were transfected with the overexpressing Prdx-2 plasmid and then treated with MPP^+^. In order to confirm the transfection efficiency, the mRNA expression of Prdx-2 was detected by RT-qPCR. According to Fig. 4A, the Prdx-2 overexpression group (OE) has significantly higher levels of Prdx-2 mRNA compared with the empty vector group (EV), the MPP^+^ group, and the control group (*P* < 0.001, n = 3). Meanwhile, the mRNA levels of Prdx-2 in the MPP^+^ group and the EV group were lower than in the control group (P < 0.01, n = 3), which is the same as with the description above. These results revealed that the plasmid was successfully transferred into the cells. Flow cytometry evaluated the ROS content in four groups. The result showed that there was a lower ROS in the OE group than in the MPP^+^ group and the EV group (*P* < 0.05, n = 3) (Fig. 4B and C), while the MPP^+^ group and the EV group had no obvious difference. The above data indicate that the overexpression of Prdx-2 reduced the accumulation of ROS in cells.

### Prdx-2 Overexpression Protected SH-SY5Y Cells from MPP^+^ Neurotoxicity

**Fig. 5:**
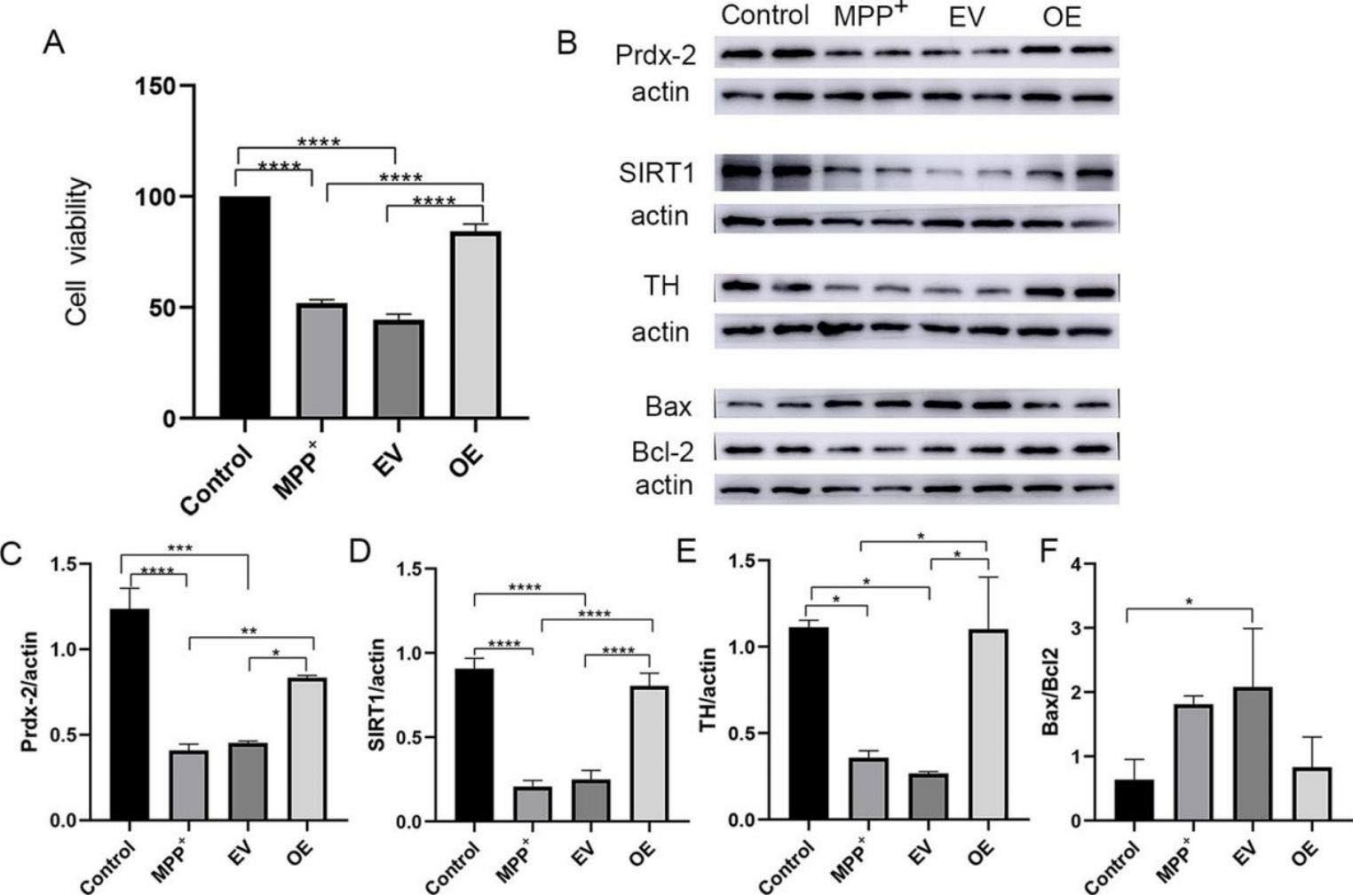
Prdx-2 overexpression protected SH-SY5Y cells from MPP^+^ neurotoxicity. (**A**) The viability of SH-SY5Y cells was analyzed by the CCK8 assay following transfection with the Prdx-2 overexpression plasmid. (**B**) Representative WB bands and protein density differences among four groups. (**C**) A densitometric analysis of protein expression of Prdx-2 among four groups. (**D**) A densitometric analysis of protein expression of SIRT1 among four groups. (**E**) A densitometric analysis of protein expression of TH among four groups. (**F**) A densitometric analysis of protein expression of Bax/Bcl-2 among four groups. *P < 0.05; **P < 0.01; ***P < 0.001; ****P < 0.0001

To determine the protection of overexpressed Prdx-2, the viability of the cells overexpressing Prdx-2 was investigated by CCK8. In the OE group, cell viability was slightly increased compared to the MPP^+^ group and the EV group (Fig. 5A). At the protein level, the WB results showed an obviously increased expression of Prdx-2 protein in the OE group, which was consistent with the RT-qPCR results (*P* < 0.05, n = 4) (Fig. 5B and C). According to Fig. 5B, D, and E, overexpression of Prdx-2 could significantly reverse the low expression of TH and SIRT1 induced by MPP^+^. Furthermore, overexpression of Prdx-2 also reversed the high ratio of Bax to Bcl-2, as shown in Fig. 5B and F. But the differences in the protein ratios of Bax and Bcl-2 among the cells in the OE group and the MPP^+^ group were not statistically significant. These findings suggest that Prdx-2 overexpression helps SH-SY5Y cells survive the MPP^+^ neurotoxic challenge, and this protective effect may be related to SIRT1.

## Discussion

Accumulating evidence has suggested that the accumulation of ROS is associated with a variety of diseases, including Alzheimer’s disease (AD), PD, and other neurodegenerative diseases that can lead to motor and cognitive dysfunction [21–23].

Meanwhile, research on antioxidant enzymes related to human neurodegenerative diseases has always attracted considerable attention, especially the Prdx family [24]. The proteomics results in our previous study showed that the Prdx-2 levels in the plasma were reduced in PD patients compared with healthy controls [12]. But studies on the mechanism of action of Prdx-2 are limited. This compelled us to learn more about the mechanism of action.

This study used MPP^+^-treated SH-SY5Y cells as the PD model in vitro. MPP^+^ was formed through MPTP, the neurotoxin, and its oxidation by monoamine oxidase-B (MAO-B) in vivo, and MPP^+^ undergoes a redox reaction in neurons, forming oxygen radicals that eventually lead to neuronal death [25]. This study found that MPP^+^ induces cell viability changes and the accumulation of ROS. The findings demonstrated that MPP^+^ has cytotoxic effects. And the toxicity level was concentration-dependent, with high MPP^+^ concentrations resulting in severe toxicity. Furthermore, MPP^+^ causes a decrease in TH and Prdx-2. The decline of TH means that the cells’ ability to synthesize dopamine is reduced. This simulates dopamine dysfunction in Parkinson’s disease patients. However, overexpression of Prdx-2 reduces MPP^+^-induced toxicity in SH-SY5Y cells, decreases the accumulation of ROS, increases cell viability, and improves the level of TH. These findings are in keeping with a previous study that found overexpression of Prdx-2 protected neurons from death after an MPP^+^ treatment, while downregulation of Prdx-2 increased oxidative stress, resulting in neuronal death. Moreover, Prdx-2 overexpression prevented the loss of dopaminergic neurons in the MPTP mouse model of PD [13]. But studies on the mechanism of action of Prdx-2 are limited. According to Hu et al. [26], Prdx-2 prevented 6-hydroxydopamine (6-OHDA)-induced dopaminergic neurodegeneration in MN9D dopamine neurons by controlling Trx’s redox status and preventing the activation of the JNK/p38 signaling pathway and ASK1 downstream. The comprehensive literature search identified SIRT1 as being in a crucial position in the signaling pathway network, and it can activate multiple signaling pathways to enable the body to exert antioxidant, anti-inflammatory, and anti-apoptotic effects [14]. This study also revealed that overexpression of Prdx-2 increased the level of SIRT1.

SIRT1 is a deacetylase, and the benefit of SIRT1 in neurodegenerative diseases was first reported by Graff [27]. There was a study that found that in SH-SY5Y cells, SIRT1 can directly deacetylated the histone residue H3K9 in the p53 promoter as a way to prevent apoptosis. In addition, resveratrol activates SIRT1 to regulate p53 to exert anti-inflammatory effects and protect against rotenone-induced dopaminergic neurodegeneration [28]. In contrast, Kitao et al. [29] used SIRT1 overexpression mice to study PD models, and SIRT1 overexpression mice did not protect against MPTP toxicity in nigrostriatal DA neurons compared to controls. This suggests that SIRT1 needs to function in both the signaling network and the deacetylase activity network to provide protection against PD pathology. However, the relationship between Prdx-2 and SIRT1 in PD has not been reported. Both Prdx-2 and SIRT1 have antioxidant effects, but the relationship between Prdx-2 and SIRT1 in PD has not been reported. The results of this study revealed that MPP^+^ reduces SIRT1 levels while Prdx-2 overexpression increases SIRT1 levels. This hints at the possibility that Prdx-2 may function in SH-SY5Y cells through the SIRT1 protein. In the study by Zhang et al. [17], the nitrosylation of Prdx2 caused the accumulation of endogenous H_2_O_2_, and then SIRT1 phosphorylated and inhibited its deacetylation activity toward p53 in A549 cells or FOXO1 in NCI-H1299 cells. The total Prdx-2 was measured in this study, but not its S-nitrosylation. This is a limitation of this research, and it is the future direction. Another limitation of this study is that the downstream signaling molecules of SIRT1 aren’t detected, such as nuclear factor E2 related factor (Nrf2), nuclear factor E2 related factor 2 (Nef2), and fork head box class O (FOXO) [15].

In addition, this study measured the levels of apoptosis-associated proteins Bax and Bcl-2. Bax aids in promoting cell death, while Bcl-2 plays a significant role in discouraging it. The ratio of Bax/Bcl-2 was usually used as a ruler to measure cell apoptosis [30, 31]. The results showed that, although there was no statistically significant difference between the MPP^+^ group and the OE group in terms of Bax/Bcl-2, there was a clear downward trend in the OE group. This reflects the fact that overexpression of Prdx-2 may reverse the increase in Bax/Bcl-2 caused by MPP^+^, which is in accordance with the results of cell viability. This similarly suggests that Prdx-2 is involved in the apoptotic process.

Taken together, the findings of the study, which include an increase in the generation of ROS, a depolarization of the mitochondrial membrane potential, and a decrease in cellular activity and the level of TH, imply that MPP^+^ is neurotoxic.

Overexpression of Prdx-2 protected SH-SY5Y cells from MPP^+^-induced toxicity by increasing SIRT-1 levels, reducing the accumulation of ROS, and decreasing the ratio of Bax/Bcl-2.

## Conclusion

The aim of this study was to demonstrate that Prdx-2 has antioxidative and cytoprotective effects in the cell model of Parkinson’s. The cell model was built with SH-SY5Y cells and MPP^+^. We demonstrated that MPP^+^ was neurotoxic by measuring the ROS content, the mitochondrial membrane potential status, the cellular activity, and the protein expression levels of TH, Prdx-2, SIRT1, Bax, and Bcl-2 in the cells. Then we repeated measurements of ROS content, cellular activity, and protein level changes in cells overexpressing Prdx-2. The results found that increased levels of Prdx-2 could diminish ROS accumulation, raise SIRT1 and TH expression, lower Bax/Bcl-2, and promote cell viability. These not only reflect the protective effect of Prdx-2 in PD but also suggest that this effect may be associated with SIRT1. This shows that improving antioxidant defense can prevent the development of PD, and Prdx-2 may be a viable target for antioxidant therapy. However, whether Prdx-2 upregulation affects SIRT1’s downstream signaling components needs to be investigated further.

## Data Availability

The datasets generated during and/or analysed during the current study are available from the corresponding author on reasonable request.
